# Regulation of nucleus‐encoded trans‐acting factors allows orthogonal fine‐tuning of multiple transgenes in the chloroplast of *Chlamydomonas reinhardtii*

**DOI:** 10.1111/pbi.14557

**Published:** 2024-12-28

**Authors:** Pawel Mateusz Mordaka, Kitty Clouston, Aleix Gorchs‐Rovira, Catherine Sutherland, Daniel Qingyang Zhang, Katrin Geisler, Payam Mehrshahi, Alison Gail Smith

**Affiliations:** ^1^ Department of Plant Sciences University of Cambridge Cambridge UK; ^2^ Present address: Institute of Quantitative Biology Biochemistry and Biotechnology, School of Biological Sciences University of Edinburgh Edinburgh UK

**Keywords:** chloroplast engineering, gene expression, vitamin‐responsive regulatory elements, metabolic engineering, diterpenoid biosynthesis

## Abstract

The green microalga *Chlamydomonas reinhardtii* is a promising host organism for the production of valuable compounds. Engineering the *Chlamydomonas* chloroplast genome offers several advantages over the nuclear genome, including targeted gene insertion, lack of silencing mechanisms, potentially higher protein production due to multiple genome copies and natural substrate abundance for metabolic engineering. Tuneable expression systems can be used to minimize competition between heterologous production and host cell viability. However, complex gene regulation and a lack of tight regulatory elements make this a challenge in the *Chlamydomonas* chloroplast. In this work, we develop two synthetic tuneable systems to control the expression of genes on the chloroplast genome, taking advantage of the properties of the vitamin B_12_‐responsive *METE* promoter and a modified thiamine (vitamin B_1_) riboswitch, along with nucleus‐encoded chloroplast‐targeted regulatory proteins NAC2 and MRL1. We demonstrate the capacity of these systems for robust, fine‐tuned control of several chloroplast transgenes, by addition of nanomolar levels of vitamins. The two systems have been combined in a single strain engineered to avoid effects on photosynthesis and are orthogonal to each other. They were then used to manipulate the production of an industrially relevant diterpenoid, casbene, by introducing and tuning expression of the coding sequence for casbene synthase, as well as regulating the metabolite flux towards casbene precursors, highlighting the utility of these systems for informing metabolic engineering approaches.

## Graphical abstract

A strain of *Chlamydomonas* has been generated that allows independent regulation of two genes in the chloroplast by addition of vitamins B_12_ and thiamine to the culture medium.

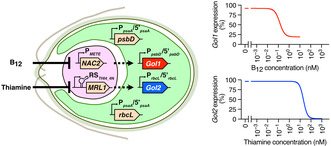

## Introduction

Microalgae are increasingly emerging as host organisms for industrial biotechnology (Fernández *et al*., 2021; Sun *et al*., 2018). Their unicellular nature and relatively fast growth enable their mass cultivation in scalable bioreactors, and their photosynthetic capacity allows direct channelling of CO_2_ towards compounds of interest, unlike conventional heterotrophic bacteria and yeast platforms, which require external carbon sources (Moses *et al*., 2017). *Chlamydomonas reinhardtii* is one of the best characterized microalgal species with a wealth of genomic, transcriptomic and metabolic information (Salomé and Merchant, 2019) and extensive tools for modification of both nuclear and organellar genomes (Georgianna and Mayfield, 2012; Scaife *et al*., 2015). Introduction of heterologous genes into the nucleus of *Chlamydomonas* has resulted in the production of proteins (e.g. Richter *et al*., 2018; Tran *et al*., 2013), biofuel precursors and platform chemicals (e.g. Kong *et al*., 2019; Yahya *et al*., 2023) and high‐value compounds (e.g. Einhaus *et al*., 2021, 2022; Wichmann *et al*., 2018).

*Chlamydomonas* cells contain a single large chloroplast with multiple copies of a prokaryote‐derived genome (plastome). The chloroplast is a biosynthetic hub, naturally abundant in many building blocks, making it an attractive site for producing a range of compounds (Jackson *et al*., 2021) and techniques for transforming the *Chlamydomonas* chloroplast are among the best characterized (Larrea‐Alvarez and Purton, 2020; Purton, 2007). Stable transformants can be generated within 4 weeks (Jackson *et al*., 2021), compared to 2–6 months in land plants (Frangedakis *et al*., 2021; Maliga *et al*., 2021). Moreover, engineering the *Chlamydomonas* chloroplast genome has several advantages over the nuclear genome. Transgenes can be efficiently targeted to specific sites in the plastome due to efficient homologous recombination (Day and Goldschmidt‐Clermont, 2011). The chloroplast lacks silencing mechanisms, resulting in greater stability of transformed lines (Bock, 2015), and contains around 80–90 plastome copies per cell (Gallaher *et al*., 2018; Misumi *et al*., 1999), enabling potentially higher heterologous protein production than from the nuclear genome (Rasala *et al*., 2011). Chloroplast engineering in *Chlamydomonas* has been used to produce metabolites (Gimpel *et al*., 2015), active pharmaceutical ingredients (Dyo and Purton, 2018), therapeutic proteins (Taunt *et al*., 2018) and chemicals (Wichmann *et al*., 2018).

In conventional industrial biotechnology hosts, such as bacteria or yeasts, regulation of heterologous gene expression is frequently included in the engineering design, to allow balance to be maintained between cell viability and heterologous production, thus minimizing competition for cellular resources. The capacity to modulate independently the expression of multiple transgenes can enable fine‐tuning of entire pathways, for instance, to optimize metabolic flux and overcome the accumulation of toxic intermediates, and sophisticated multi‐gene regulation is well established for *Escherichia coli* and yeast (Besada‐Lombana *et al*., 2018; Meyer *et al*., 2018). In contrast, most chloroplast genetic engineering in *Chlamydomonas* has involved the integration of single transgenes with a selectable marker, usually expressed constitutively (Kumar *et al*., 2020).

Resources for regulation of chloroplast gene expression in *Chlamydomonas* include inducible expression under the bacterial *lac*‐repressor regulatory system (Kato *et al*., 2007) and a cold‐inducible system known as CITRIC, which exploits a temperature‐sensitive tryptophan tRNA for translational read‐through (Young and Purton, 2018). Recently, the 5′UTR of the *psaA* transcript has been reported to act as an RNA thermometer that undergoes conformational changes in a temperature‐dependent manner, enhancing translation initiation after a shift from 25 °C to 40 °C. The 5′UTR has been further engineered by base modifications resulting in undetectable protein expression at lower temperatures (Chung *et al*., 2023).

An alternative approach to regulating the expression of chloroplast transgenes would be to take advantage of the fact that many endogenous chloroplast genes are dependent on nucleus‐encoded protein factors. For instance, the tetratricopeptide repeat (TPR) protein NAC2 (Boudreau *et al*., 2000) is essential for the expression of the chloroplast gene *psbD*, which encodes the D2 protein of photosystem II (Kuchka *et al*., 1989). NAC2 is required for correct transcript processing by binding and stabilizing target mRNA at the 5′UTR. The presence of the *psbD* 5′UTR was shown to be necessary and sufficient for the regulation of any gene sequence by NAC2 (Surzycki *et al*., 2007). By coupling nuclear *NAC2* expression to the copper‐sensitive promoter *CytC6*, copper‐induced repression was demonstrated for both *psbD* and the spectinomycin resistance gene *aadA*, when combined with *psbD* 5′UTR. A comparable system, in which NAC2 was expressed under control of the thiamine‐pyrophosphate (TPP) sensitive riboswitch from the thiazole synthase gene, *THI4* (Croft *et al*., 2007), was used to study the function of essential chloroplast genes, through thiamine‐induced downregulation (Ramundo *et al*., 2013; Ramundo and Rochaix, 2015). These studies also established NAC2‐independent expression of *psbD* (and thereby PSII) by replacing the 5′UTR of *psbD*, making NAC2 available to regulate chloroplast genes of interest selectively, without affecting its native target.

In this work, we explored the potential to combine different nuclear genetic elements to establish a set of versatile and sensitive systems to regulate chloroplast transgene expression in *Chlamydomonas*. As well as NAC2, we used MRL1, a nucleus‐encoded pentatricopeptide repeat (PPR) protein, which binds and stabilizes the Rubisco large subunit (*rbcL*) transcript at the 5′UTR, and has been shown to be essential and sufficient for the expression of its specific gene target (Johnson *et al*., 2010). These factors were regulated by either an enhanced TPP‐responsive riboswitch, *THI4*_4N (Mehrshahi *et al*., 2020) or the promoter from the *METE* gene, which encodes cobalamin‐independent methionine synthase (Croft *et al*., 2005; Helliwell *et al*., 2011) and which is repressed by cobalamin (vitamin B_12_) (Helliwell *et al*., 2014). We investigated the sensitivity of the systems to addition of the vitamin ligands. We also tested the efficacy of regulation to investigate ways to optimize production of a high‐value compound in *Chlamydomonas*, thus establishing proof‐of‐principle for sophisticated metabolic engineering of this organism, essential for it to be developed as a production platform for industrial biotechnology.

## Results

### Establishing tuneable expression of chloroplast genes via their nucleus‐encoded regulating factors

**Figure 1:**
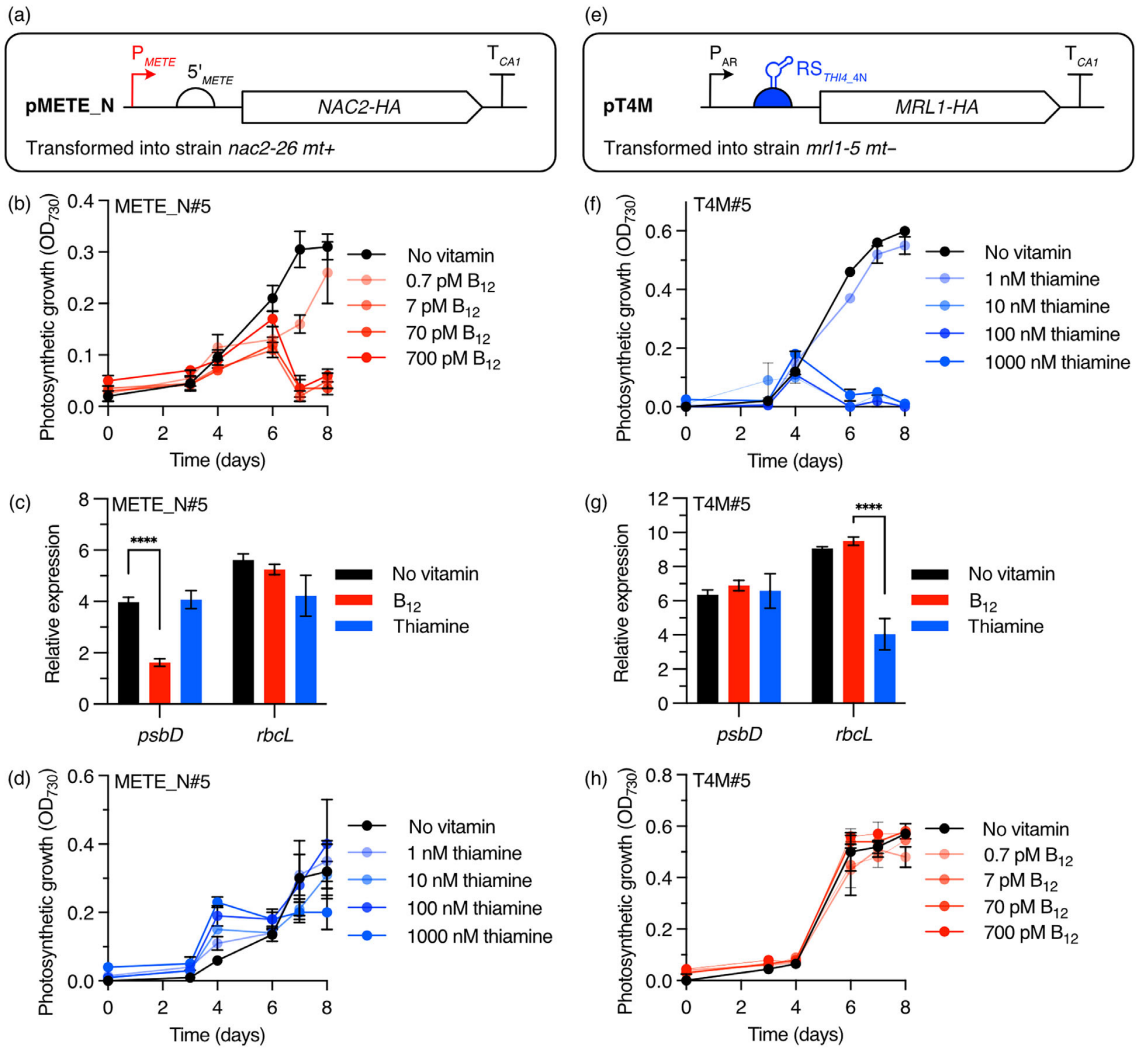
Generation of *Chlamydomonas* strains where photosynthesis can be regulated using vitamins. (a) Schematic of construct pMETE_N used to complement the *nac2‐26* strain, where NAC2 is controlled by the *METE* promoter and 5′UTR. (b) Effect of increasing concentrations of B_12_ on growth of one such complemented strain, METE_N#5. Cells were grown in photoautotrophic HSM media and growth was monitored by measuring optical density at 730 nm (OD_730_). (c) RT‐qPCR of chloroplast transcripts for *psbD* and *rbcL* genes in METE_N#5 growth in TAP media without vitamins or with 700 pM B_12_ or 1000 nM thiamine. Paired *t*‐test significance level ****P < 0.0001. (d) Photosynthetic growth of METE_N#5 in increasing concentrations of thiamine. (e) Schematic of plasmid pT4M used to complement *mrl1‐5* mutant strain where expression of MRL1 is controlled by the thiamine‐responsive riboswitch (*THI4*_4N). (f) Repression of photosynthetic growth of one representative complemented strain, T4M#5, with thiamine. (g) RT‐qPCR of *psbD* and *rbcL* transcripts in T4M#5 in TAP media without vitamins or with 700 pM B_12_ or 1000 nM thiamine. ****P < 0.0001. (h) Photosynthetic growth of T4M#5 with increasing concentrations of B_12_. Error bars represent standard deviations (*n* = 3).

*Chlamydomonas* mutant strains *nac2‐26* (Kuchka *et al*., 1989) and *mrl1‐5* (Johnson, 2011), contain nuclear genome mutations that disrupt the *NAC2* and *MRL1* genes, respectively (Table S1). This prevents stabilization of their respective target chloroplast transcripts *psbD* and *rbcL*, leading to non‐photosynthetic phenotypes. These strains were each complemented with constructs containing the wildtype gene sequences under synthetic regulation. The *NAC2* coding sequence was cloned under the control of the B_12_ repressible *METE* promoter (P_*METE*_) (Helliwell *et al*., 2014) and the resulting pMETE_N construct (Figure 1a; Table S2) was introduced by electroporation into the *nac2‐26* strain. Positive transformants were obtained by selecting for growth on minimal media, indicating successful complementation and restoration of the photosynthetic phenotype due to stabilization of the *psbD* transcript. We then tested B_12_‐mediated regulation of NAC2 in several independent transformants by growing them in minimal media without supplementation or with B_12_. The majority (65%) showed reduction in growth in response to B_12_ (Table S3), indicating effective repression of NAC2/*psbD* by P_*METE*_, which would impair production of functional photosystem II. The presence of the transgene was verified by PCR in one of the complemented lines, METE_N#5 (Figure S1a), and the response to increasing concentrations of B_12_ was determined (Figure 1b). Photosynthetic growth was significantly reduced in the presence of ≥7 pM B_12_ relative to the no‐vitamin control. Similar profiles were obtained for two other transformants (Figure S2a). There was also a significant reduction in *psbD* transcript levels determined by RT‐qPCR (Figure 1c). In contrast, no significant difference in transcript levels or photosynthetic growth was observed for the METE_N#5 line in the presence of thiamine (Figure 1c,d). Similarly, the transformation of the *mrl1‐5* mutant with the construct pT4N (Figure 1e; Table S2) encoding the *MRL1* gene under the control of *THI4_*4N riboswitch (RS_*T4*_) allowed selection for restoration of photosynthetic capacity. More than 90% of transformants responded to thiamine supplementation (Table S3). After verifying the presence of the transgene in one line, T4M#5 (Figure S1b), a more detailed analysis of this line showed that ≥10 nM thiamine was sufficient to reduce growth (Figure 1f and Figure S2b for two other transformants) and transcript levels for *rbcL* (Figure 1g), but B_12_ had no effect (Figure 1g,h). Together these data indicate that these systems are effective and independent of one another and that photosynthetic growth can be used as a proxy for regulation of *rbcL* or *psbD* expression.

**Figure 2:**
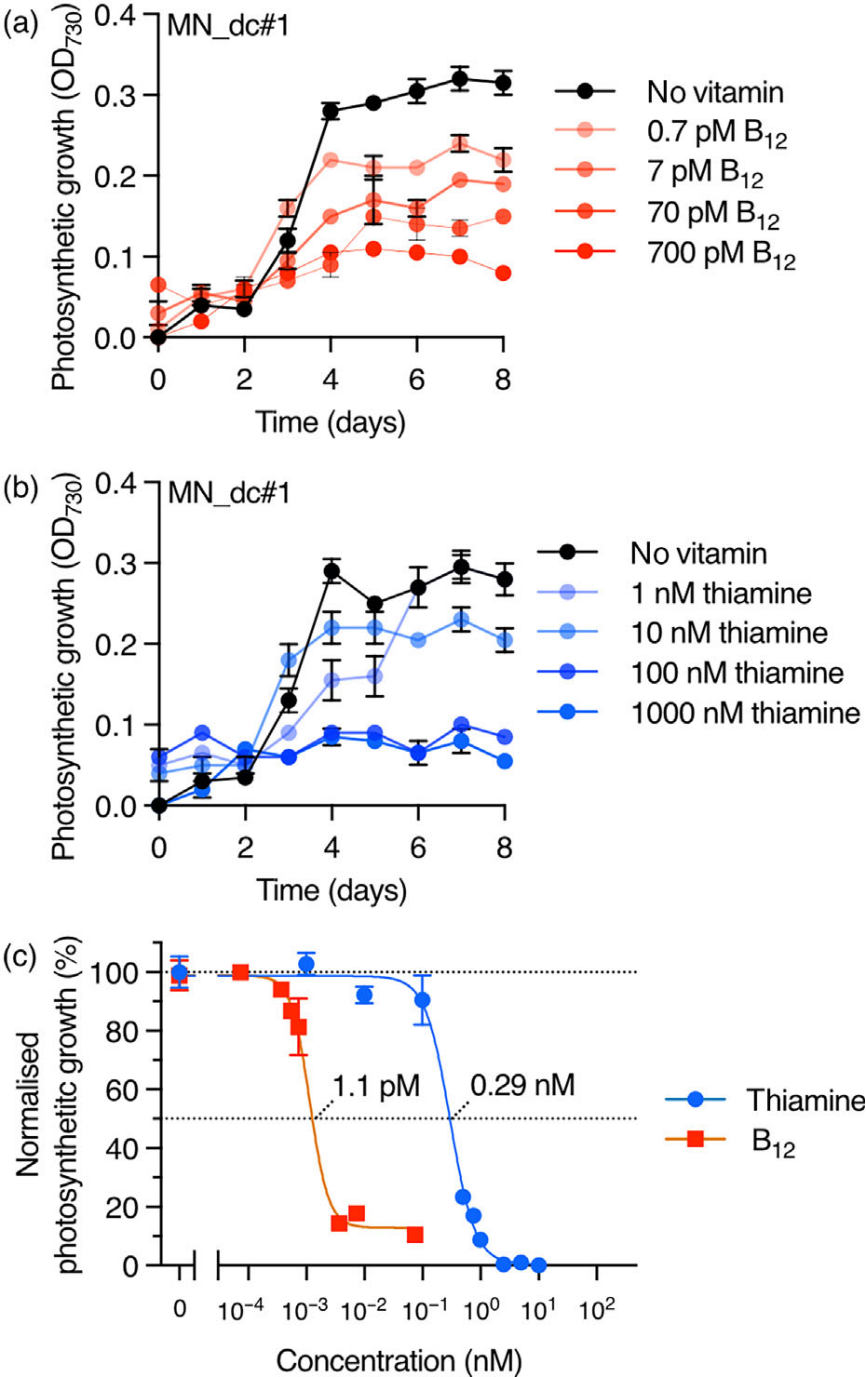
Combining P_*METE*_‐NAC2 and RS_*T4*_‐MRL1 systems in one strain. Strains T4M#5 and METE_N#5 were crossed to generate the double‐complemented, double‐mutant strain, MN_dc#1. Effect of increasing concentrations of B_12_ (a) and thiamine (b) on photosynthetic growth of MN_dc#1 measured by OD_730_ (c) Sigmoidal dose–response curves were used to estimate ED_50_ values on day 5 (1.1 pM for B_12_ and 0.29 nM for thiamine). Error bars represent standard deviations (*n* = 3).

We took advantage of having both mating types for the two original mutant strains (Table S1) and crossed two of the complemented strains, T4M#5 (mt−) and METE_N#5 (mt+). We identified a single strain (called MN_dc#1) that was a double *mrl1*, *nac2* mutant containing both transgenes, as verified by PCR (Figure S1c). Addition of either B_12_ (Figure 2a) or thiamine (Figure 2b) repressed the growth of MN_dc#1 in photoautotrophic media, indicating both regulatory systems were functional in this strain. To characterize the vitamin responsiveness of each system, we used a four‐parameter log‐logistic model (Mehrshahi *et al*., 2020), typical of dose–response analysis, to assess relative photosynthetic growth as a function of vitamin concentration and thus determine the effective dose required for 50% response (ED_50_) and the maximum repression capacity, the percentage reduction in response relative to the positive control samples. The data fitted the expected sigmoidal dose response curve (Figure 2c). Under these conditions, ED_50_ values on day 5 were determined to be 1.1 pM for B_12_ and 0.29 nM for thiamine. It should be noted that full repression of growth was achieved with the highest concentrations of thiamine, whereas residual growth was always observed with B_12_. Nonetheless, strain MN_dc#1 contained two independent regulatable systems that enable vitamin‐mediated fine‐tuning of chloroplast gene expression, via nucleus‐encoded factors and regulatory elements.

### Uncoupling native chloroplast genes from synthetic regulation

**Figure 3:**
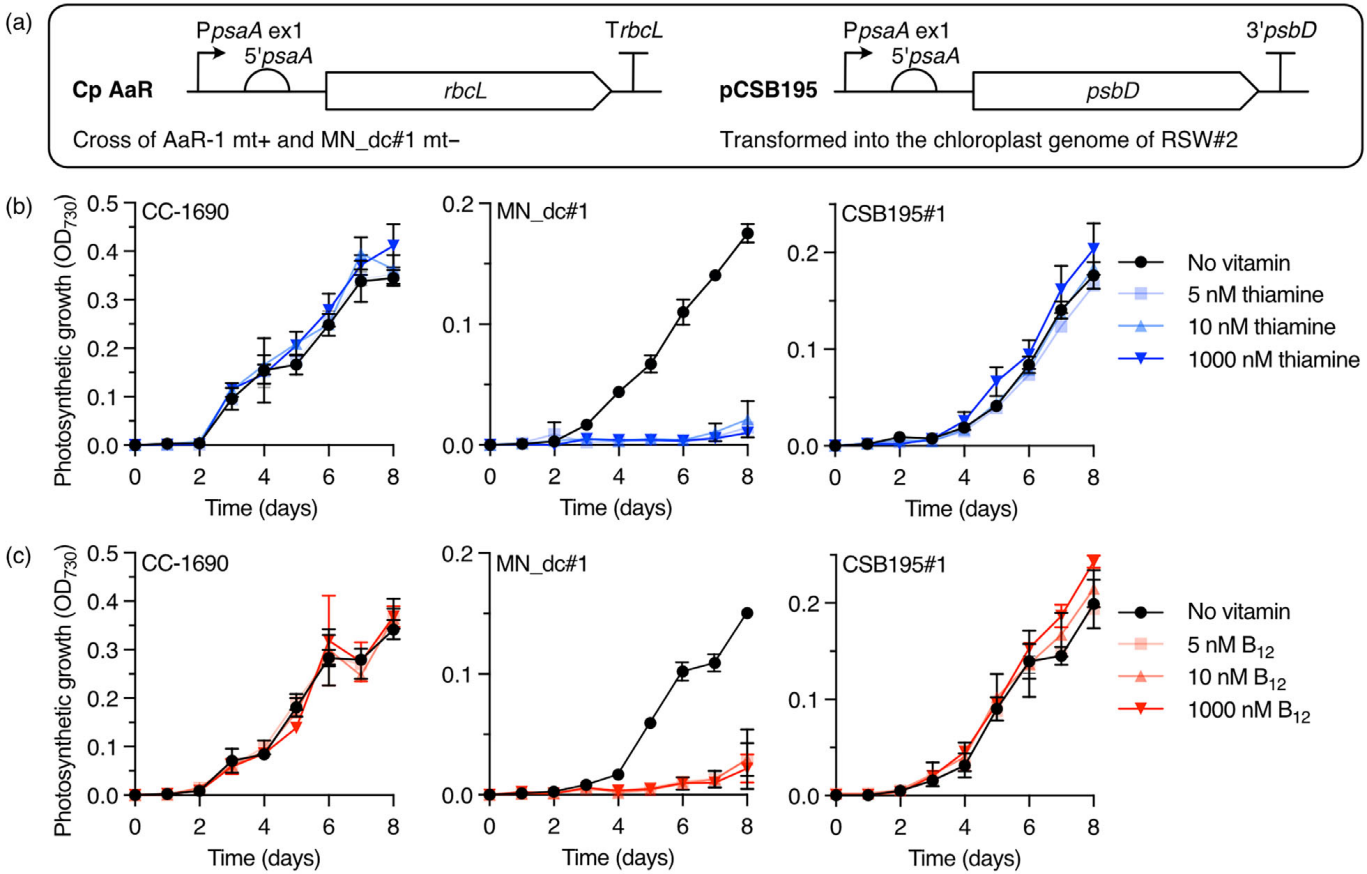
Decoupling photosynthesis from MRL1 and NAC2 regulation. (a) Schematic showing the genetic changes to generate strain CSB195 from MN_dc#1. The promoter and 5′UTR of *rbcL* were replaced with the promoter and 5′UTR of *psaA* by crossing strain MN_dc#1 mt− with AaR‐1 mt+ to generate strain RSW#2. The promoter and 5′UTR of *psbD* were replaced by those for *psaA* by chloroplast transformation of this strain with plasmid pCSB195, using flanking sequences to allow homologous recombination at that locus (see Table S2 for details) and a positive–negative marker gene, *codA*.*aadA*, to select for transformants and subsequently delete the marker. Effect of increasing concentrations of thiamine (b) or B_12_ (c) on photosynthetic growth in HSM as measured by OD_730_ of the engineered strains, and a wild‐type strain CC‐1690 for comparison. Error bars represent standard deviations (*n* = 3).

Fusing the target 5′UTRs of NAC2 (i.e. from *psbD*) or MRL1 (from *rbcL*) upstream of transgenes in the chloroplast genome in the engineered *Chlamydomonas* strain MN_dc#1 should allow their expression to be regulated by B_12_ or thiamine, respectively. To ensure that this did not interfere with expression of the native *psbD* and *rbcL* genes, it was necessary to change their 5′UTRs to uncouple them from NAC2 and MRL1 regulation. Strain AaR‐1 (Table S1), obtained from Dr. Katia Wostrikoff (Institute of Physical and Chemical Biology, Paris), had been engineered so that the native *rbcL* promoter and 5′UTR were replaced with those from *psaA* (schematic shown in Figure 3a), which is expressed independently of MRL1. AaR‐1 (mt+) was crossed with MN_dc#1 (which was mt−, Figure S1c) and progeny identified that contained the same mutations as MN_dc#1, namely the complementing RS_*T4*_‐MRL1 and P_*METE*_‐NAC2 (Figure S3a), and the *rbcL* coding sequence with the *psaA* promoter and 5′UTR. Photosynthetic growth of the resulting strain, called RSW#2, was responsive to B_12_ but not thiamine (Figure S3b), indicating expression of functional RbcL with the *psaA* promoter and 5′UTR, that is, it was no longer influenced by MRL1. Surzycki *et al*. (2007) and Ramundo *et al*. (2013) successfully replaced the 5′UTR of *psbD* with that of *petA* and *psaA*, respectively, making expression of *psbD* independent of NAC2. Using a similar approach, we transformed the chloroplast of strain RSW#2 with constructs containing the *psbD* coding sequence with either *petA* or *psaA* promoter and 5′UTRs to generate strains CSB193 and CSB195, respectively. Chloroplast transformation was performed by biolistic bombardment, using antibiotic selection to identify transformants. The constructs contained the positive–negative *codA‐aadA* selection marker (Jackson *et al*., 2022) and flanking sequences for homologous recombination in the chloroplast at the *psbD* locus. Transformants were selected in the presence of spectinomycin and after reaching homoplasmy (Figure S4a), the selectable marker was removed by counterselection with 5‐fluorocytosine (Jackson *et al*., 2022), allowing the strains to be transformed again. Although exchanging the endogenous promoter‐5′UTR for *petA* allowed photosynthetic growth in CSB193 transformants, it was less robust than the parental strain, RSW#2, whereas there was no deleterious effect with the *psaA* promoter‐5′UTR (Figure S4b) within CSB195 transformants, so one of these was taken forward.

Assessment of photosynthetic growth of a representative homoplasmic transformant, CSB195#1, alongside MN_dc#1 and a wildtype (WT) strain CC‐1690 in the presence and absence of B_12_ and thiamine, showed that growth of MN_dc#1 was responsive to both vitamins (Figure 3b,c), whereas neither CC‐1690 nor CSB195#1 showed any significant change in growth in the presence of either vitamin. This confirms the expression of functional RbcL and PsbD under the *psaA* promoter and 5′UTR in CSB195#1, independent of regulation by MRL1 and NAC2. This strain contains several genetic modifications and the growth was seen to be slightly slower than that of an unmodified WT strain, CC‐1690, in this experiment (Figure 3b,c). We investigated this further by extended culturing in both photoautotrophic media (HSM) and mixotrophic (TAP) media (Figure S5). There was little indication of growth impairment in CSB195#1 and even a slight increase in growth rate in TAP.

### Using the repressible systems to regulate reporter genes in the chloroplast

**Figure 4:**
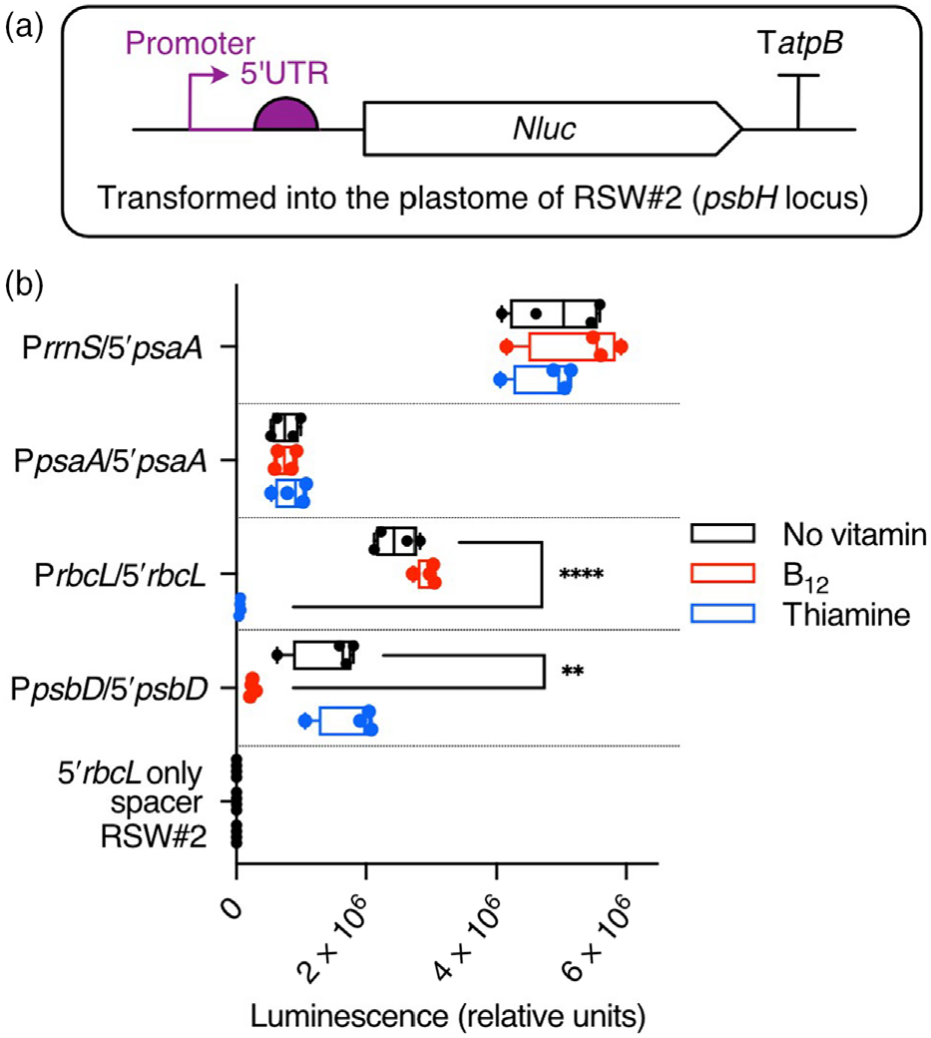
Testing native chloroplast promoter and 5′UTR combinations for responsiveness to the nuclear factors. (a) Schematic of the construct design where the reporter protein Nanoluciferase is controlled by different combinations of chloroplast promoters and 5′UTRs (Table S2). The expression cassettes were inserted into the single copy *psbH* locus in the chloroplast genome of RSW#2. (b) Relative activity of luciferase in the transgenic strains. Independent transformants of each construct (*n* = 4) were grown in a TAP medium and samples were harvested on day 4. To test the regulation of reporter expression, cultures were supplemented with B_12_ (10 nM) or thiamine (1 μM).

To characterize these systems in more detail, we used the NanoLuc^®^ Luciferase (*Nluc*) gene as a reporter (Hall *et al*., 2012). It was codon optimized for the *Chlamydomonas* chloroplast and cloned downstream of the promoter/5′UTRs of *rbcL* and *psbD* together with other commonly used promoters/5′UTR combinations (Table S2). These were introduced independently into the single copy *psbH* locus in the chloroplast genome of RSW#2 using the *codA.aadA* selectable marker (Figure 4a). After several subcultures to reach homoplasmy, four independent transformants for each construct were grown in a TAP medium with and without vitamins, and luciferase activity was measured on day 4. The lowest activity was seen for those with *psaA* promoter/5′UTR (pCSB182 in Table S2), whereas adding the promoter of 16S rRNA gene (P*rrnS*) to the *psaA* 5′UTR (pCSB181) raised the activity 5‐fold (Figure 4b). Neither was affected by thiamine or B_12_ addition. The activities driven by the *psbD* (pCSB200) and *rbcL* promoter/5′UTRs (pCSB183) were ~1.7 and 2.5 times that of *psaA*, respectively. In both cases, luciferase activity was repressed by the addition of the cognate vitamin but unaffected by the other. One further construct was investigated, where the 16S promoter was combined with the *rbcL* 5′UTR (pCSB186). Although this retained its responsiveness to thiamine, the activity under the permissive condition was reduced by about 60% (Figure S6). These results indicate that the overall level of gene expression driven by the different parts is not additive. Nonetheless, they confirm that the two regulatory systems, P_*METE*_‐NAC2/*psbD* and RS_*T4*_‐MRL1/*rbcL*, can operate independently of one another, in other words, are orthogonal.

**Figure 5:**
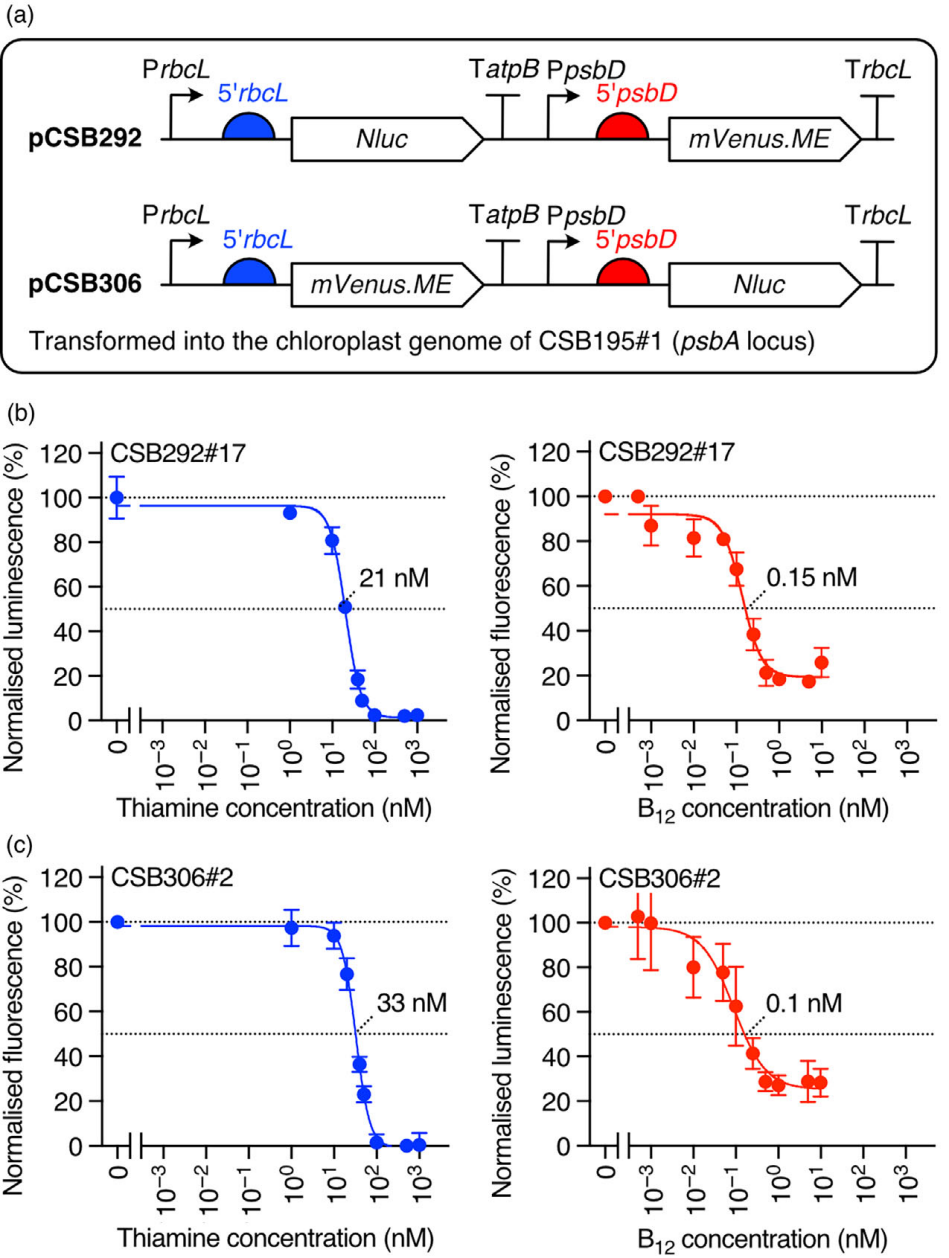
Characterizing the P_*METE*_‐NAC2 and RS_*T4*_‐MRL1 systems using two reporter proteins. (a) Schematic of reporter constructs pCSB292 and pCSB306 encoding Nanoluciferase and mVenus.ME is controlled by *rbcL* and *psbD* promoters and 5′UTRs. These were used to transform the chloroplast genome of CSB195#1 in the double‐copy *psbA* locus. (b) ED_50_ for thiamine and B_12_ repression of *Nluc* and *mVenus.ME* expression, respectively, in a representative transformant of pCSB292. (c) ED_50_ for thiamine and B_12_ repression of *mVenus.ME* and *Nluc* expression in a representative transformant of pCSB306. Error bars represent standard deviations (*n* = 3).

To characterize further the orthogonality and vitamin‐responsiveness of the regulatable systems, we introduced reporters into CSB195#1, Nluc to provide quantitative measurements of transgene expression levels at a single time point, and the mVenus.ME fluorescent protein (Jackson *et al*., 2022), which offers easy monitoring of protein expression over several days. These reporters would provide a more direct readout of protein expression levels under vitamin regulation compared to measuring photosynthetic growth in MN_dc#1 and would thereby enable robust analysis of system dynamics. To achieve this, two multi‐gene constructs were generated (Figure 5a). pCSB292 encoded *NLuc* with the *rbcL* promoter and 5′UTR (so under the control of RS_*T4*_‐MRL1) and *mVenus.ME* with the *psbD* promoter and 5′UTR (so regulated by P_*METE*_‐NAC2). Plasmid pCSB306 encoded *mVenus.ME* and *Nluc* with the reciprocal regulation. Each was transformed into the chloroplast genome of strain CSB195#1 at the *psbA* locus, which is present in two copies in the genome (Jackson *et al*., 2022), and homoplasmic lines obtained by antibiotic selection (Figure S7a). Functional expression of both transgenes was confirmed in the resulting transformants CSB292#17 and CSB306#2 (Figure S7b).

These lines were cultured in TAP media at a range of concentrations of each vitamin as before. Daily measurements of mVenus.ME fluorescence, chlorophyll autofluorescence and OD_730_ were taken and a luciferase assay was performed on day 5, estimated to be the peak in transgene expression (Figure S7c). To allow comparisons between the different conditions, the values were expressed as a percentage of that measured with no vitamins. The data were fitted to a 4‐parameter log‐logistic model (Mehrshahi *et al*., 2020), enabling determination of the ED_50_, maximum repression capacity and the dynamic range. For both reporters under both systems, the data fitted the expected sigmoidal dose–response curve (Figure 5b,c). For the RS_*T4*_‐MRL1 system, estimated ED_50_ values of 20–30 nM thiamine were determined, and approximately 0.1 nM B_12_ with the P_*METE*_‐NAC2 system. These values are in the same range as those observed previously for nuclear transgenes under the control of either the RS_*T4*_ (Mehrshahi *et al*., 2020) or P_*METE*_ (Helliwell *et al*., 2014), indicating that the regulation of the chloroplast genes is a direct reflection of the expression of the PPR/TPR genes in the nucleus.

Under the RS_*T4*_‐MRL1/*rbcL* system, full repression of reporter output to background levels was achieved, whereas with P_*METE*_‐NAC2/psbD system, a persistent basal level of expression was observed even at the highest concentration of B_12_, with a maximum repression capacity of 80% relative to the no‐vitamin control. This was similar to the effect of B_12_ on photosynthetic growth in MN_dc#1 (Figure 2). To identify which component in our artificial genetic system was responsible for this phenotype, we generated transformants in which MRL1 was regulated by P_*METE*_ (METE_M), enabling B_12_‐regulation of *rbcL* expression. Photosynthetic growth of METE_M was reduced but not fully repressed at high concentrations of B_12_, relative to the no‐vitamin condition (Figure S8). This suggests that incomplete repression via P_*METE*_, rather than NAC2, is the reason for these results.

### Controlled production of a diterpene in the *Chlamydomonas* chloroplast

**Figure 6:**
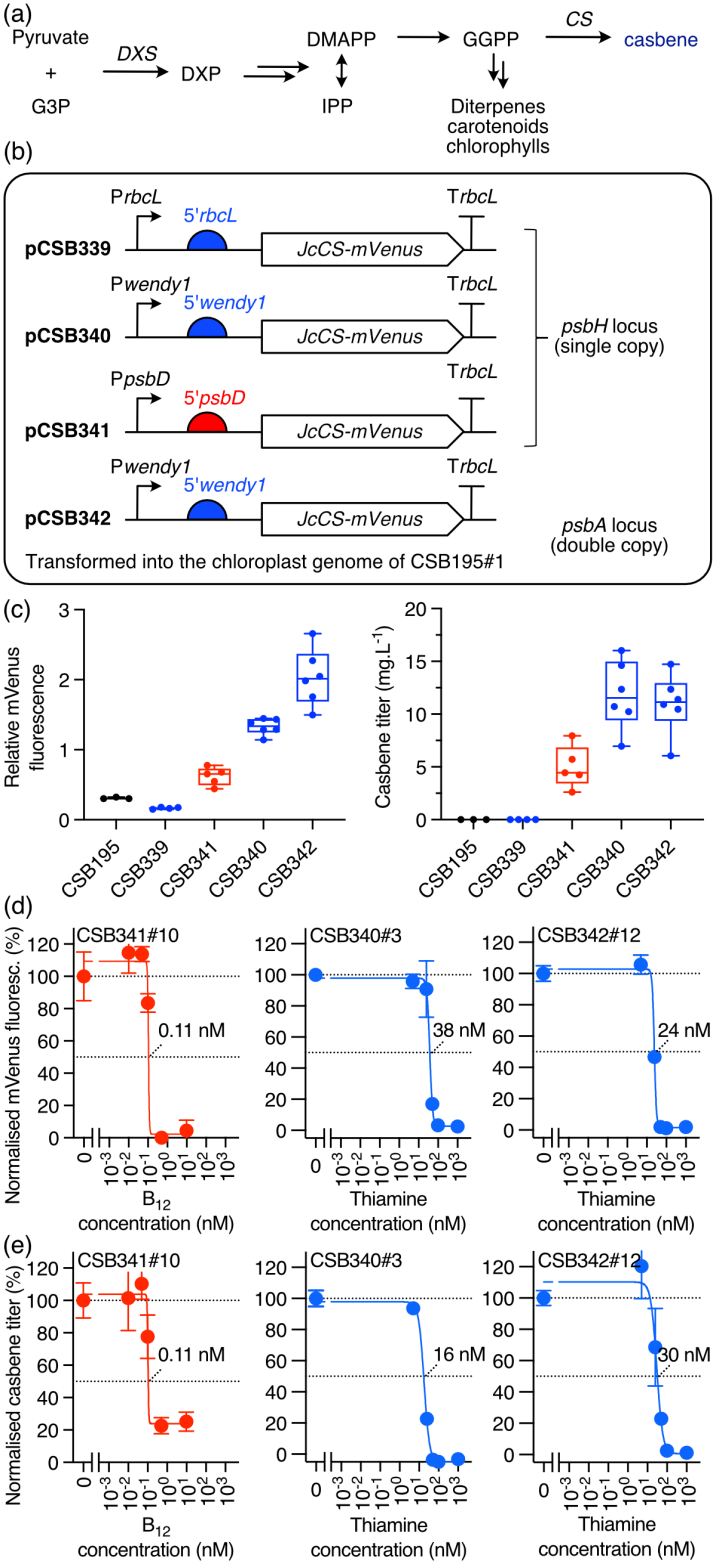
Expression and regulation of casbene synthase in the chloroplast. (a) Biosynthetic pathway of the plant natural product casbene. CS, casbene synthase; DMAPP, dimethylallyl pyrophosphate; DXP, 1‐deoxy‐d‐xylulose 5‐phosphate; DXS, 1‐deoxy‐D‐xylulose‐5‐phosphate synthase; G3P, glyceraldehyde 3‐phosphate; GGPP, geranylgeranyl pyrophosphate; IPP, isopentenyl pyrophosphate. (b) Constructs encoding CS from *Jatropha curca* fused to mVenus (*JcCS‐mVenus*) controlled by the *rbcL* (pCSB339), *wendy1* (pCSB340) or *psbD* (pCSB341) promoters and 5′UTRs were targeted into the single copy *psbH* locus. Construct pCSB342 encoding *JcCS‐mVenus* controlled by *wendy1* promoter and 5′UTR was integrated in the double copy *psbA* locus. (c) mVenus fluorescence on day 5 (left) and casbene production after 12 days (right) growth in a TAP medium of several independent homoplasmic lines of CSB339, CSB341, CSB340 and CSB342 (*n* = 4, 5, 6 and 6, respectively). Effect of increasing concentrations of B_12_ or thiamine on mVenus fluorescence (d) and casbene production (e) in lines CSB341#10, CSB340#3 and CSB342#12. Error bars represent standard deviations (*n* = 3).

The ultimate goal of developing these regulatable systems is to facilitate metabolic engineering of *Chlamydomonas* for the production of high‐value compounds. Being able to control the expression of heterologous genes encoding relevant enzymes for their biosynthesis could facilitate determining rate limiting steps, as well as mitigate potential detrimental impacts on metabolism and cell viability during the growth of the cultures. We therefore investigated the capacity of the systems to regulate a gene encoding casbene synthase, responsible for the synthesis of the diterpenoid casbene (Figure 6a), which is a precursor to certain pharmaceutical molecules (Durán‐Peña *et al*., 2014). Previously, the enzymes from plants *Ricinus communis* (Lauersen *et al*., 2018) and *Jatropha curca* (*JcCS*) (Mehrshahi *et al*., 2020) were successfully expressed in *Chlamydomonas* from the nuclear genome. Constructs were made in which the *JcCS* coding sequence tagged with mVenus and codon‐optimized for expression in the *Chlamydomonas* chloroplast, was cloned behind the promoter and 5′UTR of *rbcL* (pCSB339) for regulation by RS_*T4*_‐MRL1, or *psbD* (pCSB341), for regulation by P_*METE*_‐NAC2 (Figure 5b). In addition, a third construct (pCSB340) was made using the promoter and 5′UTR from the chloroplast‐encoded *wendy1* gene (*w1*), a gene of unknown function (Fan *et al*., 1995). The reason behind this was that the *w1* 5′UTR shares sequence similarity with that from *rbcL* (Figure S9) and is likely to be bound by MRL1. Using this rather than *rbcL* might reduce any potential interference with the expression of the latter. These three constructs contained homologous flanking regions targeting the transgene cassette for integration into the single copy *psbH* locus.

After introduction into CSB195#1 and selection of homoplasmic transformants (Figure S10a), mVenus fluorescence and casbene production were measured in cultures after 4 days and 12 days, respectively, in several independent transformants per strain. Lines encoding *JcCS‐mVenus* under the *rbcL* promoter/5′UTR (CSB339) did not produce a detectable fluorescence signal above the background seen in the parental CSB195#1 strain (Figure 6c), and no casbene peak was visible in the chromatogram for any transformant analysed. The reason for this is unknown but may have been due to incompatibility of the *JcCS* coding sequence in the context of the translation start site, known to be important for chloroplast translation (Weiner *et al*., 2020). In contrast, the strains with either *psbD* or *w1* promoter/5′UTR showed both mVenus fluorescence and detectable amounts of casbene, with mean levels of 4.8 and 12 mg/L, respectively. Based on these results, a fourth construct was generated (pCSB342) where *JcCS* was under the *w1* promoter/5′UTR but with flanking regions targeting the *psbA* locus, which is double‐copy due to its location in the inverted repeat region of the genome. We hypothesized that this might increase gene transcription and protein production compared to a single copy locus, and indeed, a 1.5‐fold higher mVenus fluorescence was observed (Figure 6c). However, this did not result in increased casbene titre.

We then tested whether the *JcCS‐mVenus* gene could be regulated under each system and explored how this impacted casbene production. A single transformant line from each strain was cultivated in TAP media supplemented with different concentrations of either B_12_ (CSB341) or thiamine (CSB340, CSB342). mVenus fluorescence and casbene production were quantified as above. A reduction in mVenus signal with increasing concentrations of vitamin was observed under both systems (Figure 6d), with a corresponding reduction in casbene titre (Figure 6e). Under the RS_*T4*_‐MRL1 system, the mVenus signal was repressed to background levels in the presence of ≥50 nM thiamine. Although a small casbene peak could be detected in the chromatogram under all conditions, the titres were 0.02 mg L^−1^ or less, 1%–2% of that in permissive conditions. When *JcCS‐mVenus* was under the control of the P_*METE*_‐NAC2 system, the mVenus signal was repressed to around 5% of the maximum in the presence of ≥0.5 nM B_12_ and that of casbene to ~20% of the maximum. The relative repressions of *JcCS‐mVenus* under the two systems, and the estimated ED_50_ values, were thus similar to those seen with the *mVenus.ME* and *Nluc* reporter genes (Figure 5b,c).

### Applying the systems to understand pathway flux towards casbene

Our systems were designed to enable the independent regulation of two transgenes in a single strain. We sought to exploit this capacity to test the engineering of the pathway towards casbene, by generating and analysing strains in which two pathway enzymes were expressed under the RS_*T4*_‐MRL1 and P_*METE*_‐NAC2 systems, respectively. Increased expression of casbene synthase (Figure 6c) had not resulted in a further increase in casbene production (Figure 6d), suggesting that the precursor may be limiting, so we chose to introduce an extra copy of 1‐deoxy‐D‐xylulose 5‐phosphate synthase (DXS), an enzyme in the MEP pathway (Figure 6a), under synthetic regulation, in addition to the endogenous enzyme. This approach has been shown to increase titres of other diterpenoids, manoyl oxide and sclareol (Einhaus *et al*., 2022; Lauersen *et al*., 2018), suggesting this could feasibly increase flux towards casbene.

**Figure 7:**
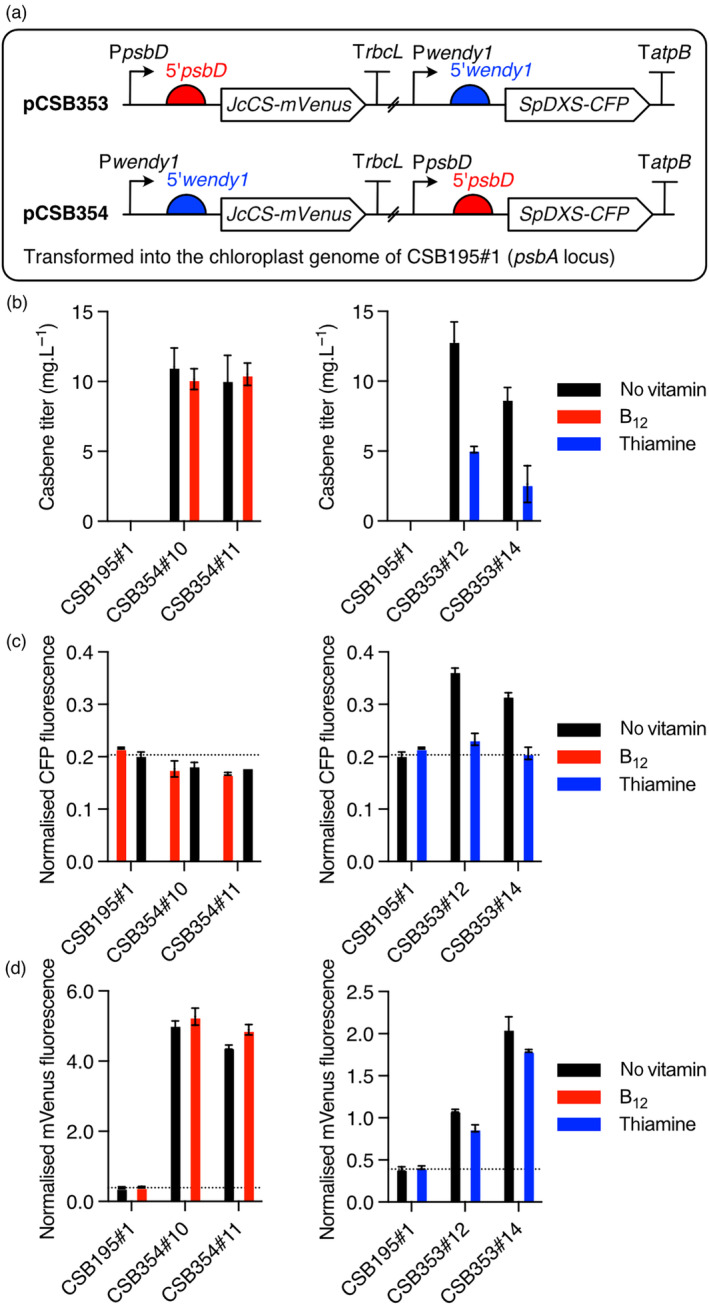
Regulation of the metabolite flux towards casbene. (a) The effect of increasing flux of the precursor to isoprenoids was investigated by introducing a second plant enzyme encoding DXS from *Salvia pomifera* (*SpDXS*). Two constructs were made, pCSB353 encoding *JcCS‐mVenus* controlled by the *psbD* promoter and 5′UTR, together with *SpDXS‐CFP* controlled by the *wendy1* promoter and 5′UTR, and the reciprocal, pCSB354. Both constructs were targeted to the double‐copy *psbA* locus. (b) Casbene production in two representative transformants of each construct after growth for 12 days in a TAP medium without vitamins or with 10 nM B_12_ (red) or 1 mM thiamine (blue). (c) CFP fluorescence on day 5 in the different transformants as a proxy for *SpDXS* expression. (d) mVenus fluorescence on day 5 in the different transformants as a proxy for *JcCS* expression. The background strain CSB195#1 was grown alongside as a control. CFP and mVenus signals were normalized to chlorophyll autofluorescence.

The DXS coding sequence from *Salvia pomifera* (*SpDXS*) tagged with CFP was codon optimized and cloned under control of the RS_*T4*_‐MRL1/*w1* system in a multi‐gene construct with *JcCS‐mVenus* under the control of P_*METE*_‐NAC2/*psbD* system (pCSB353; Figure 7a). Construct pCSB354 was the reciprocal version. Both constructs were targeted to the double‐copy *psbA* locus in CSB195#1. After transformation and selection of homoplasmic lines (Figure S10b), two independent transformants for each construct were taken for further analysis. Measurement of casbene in CSB354 transformants, cultured for 12 days in TAP without vitamins (Figure 7b, black bars), showed ~10 mg L^−1^, similar to those in CSB340 lines (Figure 6c). Since they both encoded the same *JcCS‐mVenus* gene using the *w1* promoter/5′UTR, this would imply that either DXS was not expressed in CSB354 or that the enzyme was not limiting for casbene production in this context. The former possibility was most likely since no CFP fluorescence above the background was observed in CSB354 lines (Figure 7c) and the addition of B_12_ did not alter either parameter (red bars). Conversely, in CSB353 lines containing *SpDXS‐CFP* gene under the control of the *w1* promoter/5′UTR, there was an approximately twofold increase in casbene titre compared to the CSB341 lines, where casbene synthase was expressed from the *psbD* promoter/5′UTR. The role of DXS in boosting production levels was confirmed by the addition of thiamine, which reduced casbene to the same titre as in CSB340 lines and CFP fluorescence (as a proxy for the enzyme protein) to background levels. In contrast, mVenus fluorescence as a proxy for CS expression is essentially unaffected by vitamin supplementation (Figure 7d).

## Discussion

In this work, we used several nuclear‐ and chloroplast‐genome‐encoded genetic elements and combined them to generate novel synthetic systems that allowed the independent regulation of two chloroplast transgenes in a single strain of *Chlamydomonas*. We took advantage of the properties of the vitamin B_12_‐responsive *METE* promoter (Helliwell *et al*., 2014) and a modified thiamine riboswitch (Mehrshahi *et al*., 2020), along with regulatory proteins NAC2 and MRL1. By placing the *NAC2* gene under the control of P_*METE*_, and *MRL1* under RS_*T4*_, we demonstrated selective repression of chloroplast reporter genes containing NAC2‐ and MRL1‐dependent 5′UTRs, by addition of B_12_ and thiamine, respectively (Figures 4 and 5). We also demonstrated the capacity of this system for robust, fine‐tuning of multiple chloroplast‐localized transgenes, both the endogenous *psbD* and *rbcL* genes and introduced transgenes. Finally, in strain CSB195#1, we could uncouple photosynthesis from the regulatory systems by swopping the promoter/5′UTRs from *psbD* and *rbcL* with that of *psaA*; this demonstrated that three crucial photosynthetic genes could be expressed with identical promoter/5′UTRs at the same time. We then used our systems to produce the industrially relevant diterpenoid casbene by overexpression and tuning of casbene synthase (Figure 6) and showed that the yield of the product can be regulated by subsequent regulation of the carbon flux towards the product through the MEP pathway, responsible for the synthesis of the casbene precursor (Figure 7). These results highlight the utility of being able to fine‐tune gene expression to inform metabolic engineering approaches. Lastly, all the strains used in this study possessed a cell wall and can be crossed easily, unlike the cell‐wall deficient strains of *Chlamydomonas* commonly used for nuclear genome engineering.

Regulation of transgene expression has the potential to balance cell growth and heterologous product formation. In the *Chlamydomonas* chloroplast, several gene regulation systems have been developed, including the *lac*‐repressor (Kato *et al*., 2007), CITRIC (Young and Purton, 2018) and the RNA thermometer (Chung *et al*., 2023). However, none of these offers the possibility of controlling expression of two different plastome‐integrated transgenes independently of one other in a single strain at the same time. In contrast, the P_*METE*_‐NAC2‐ and RS_*T4*_‐MRL1‐regulated transgenes can be controlled independently of one another (Figure 5), and the nucleus‐encoded trans‐acting factors are also functionally orthogonal (Figure 1). Although NAC2 has previously been shown to be regulatable by an unmodified *THI4* riboswitch and used to repress expression of chloroplast genes with a *psbD* 5′UTR (Ramundo *et al*., 2013; Ramundo and Rochaix, 2015), this is the first demonstration of using MRL1 in this way. MRL1 can bind and stabilize at least two different chloroplast transcripts, *rbcL* and *wendy1*, as using their respective promoter/5′UTR showed repression of a reporter gene in response to addition of thiamine to the growth medium (Figure 6c). Since the *mrl1‐5* strain does not express MRL1 (Johnson, 2011), and MRL1 is essential for stabilization of the *wendy1* transcript, this gene can be inferred not to play an essential role in the chloroplast under heterotrophic growth conditions. Moreover, the replacement of the *rbcL* promoter and 5′UTR in MN_dc#1 with the promoter and 5′UTR of the *psaA* gene in RSW#2 was sufficient to restore phototrophic growth of *Chlamydomonas* in the presence of thiamine (Figure S3), which suggests that *wendy1* is not involved in photosynthesis either.

Previous work by Ramundo *et al*. (2013) regulated the expression of WT NAC2 in a *nac2‐26* background, with the aim to test the effect of suppressing the expression of essential genes and determine their roles in the chloroplast. They used a combination of the unmodified *THI4* riboswitch (Croft *et al*., 2007) and approximately 1.2 kb of the region upstream of the *METE* gene, which was presumed to contain the promoter, but in fact was missing 12 bp before the *METE* transcription start site and also extended into the upstream gene. There was effective regulation with thiamine via the riboswitch, as shown by complete loss of Fv/Fm after 48 h. However, addition of B_12_ resulted in just a 40% reduction in Fv/Fm, even at 1 mg/L, indicating that the element they used from P_*METE*_ was not sufficient to repress NAC2 completely. In our case, we took a sequence of 485 bp of METE promoter and 89 bp of METE 5′UTR up to the start codon and saw much more effective control, with up to 85% repression at 1 μg/L B_12_. Basal levels of expression were nonetheless observed with all the transgenes controlled by P_*METE*_ in this study. Repression of the native *METE* gene by B_12_ is ~50‐fold compared to conditions without the vitamin and no detectable enzyme protein is present (Helliwell *et al*., 2014), suggesting that in addition to the *METE* promoter/5′UTR there are additional control elements in the *METE* gene (Sayer *et al*., 2023).

The major outcome of our work is the ability to interrogate gene expression in the chloroplast in a controlled and quantitative fashion. It has obvious applicability to metabolic engineering, where the capacity of the chloroplast genomes offers high enzyme expression and stability (Oey *et al*., 2009). By manipulating regulation and varying cassette copy numbers, the system demonstrated utility in identifying bottlenecks, which were found to be transgene expression and pathway flux (Figure 7). There was no effect of positioning the transgenes at two different loci within the chloroplast genome either at the level of expression or their ability to be regulated, but when inserted into the inverted repeat, so present as two copies, the reporter gene activity was increased (Figure 6c). In contrast, this did not result in more casbene production (Figure 7b), so while in principle higher expression of a transgene may be achieved with increasing copy number, this might not be relevant for an enzyme if the substrate is limiting. Nonetheless, the effect of different target sites on chloroplast transgene expression is relatively unexplored in the literature, and it would be of interest to determine the tolerance of the plastome to the introduction of more than one transgene, since this is likely to be necessary for any extensive metabolic engineering designs.

Regulatable expression systems also offer the means to express genes that place a metabolic burden on the cell or are even toxic. Although in this work, maximal expression of CS and DXS did not show any negative effect on the growth of the recombinant strains, deleterious effects have been reported in other systems. For instance, efforts to produce polyhydroxyalkanoates as precursors for bioplastics by constitutive expression of the PHB operon in the chloroplast of *Nicotiana tabacum* caused growth reduction and pollen sterility (Lössl *et al*., 2003), most likely due to the impact on central metabolism. Similarly, constitutive production of antimicrobial peptides resulted in severe growth defects (Hoelscher *et al*., 2022). However, the authors overcame this by cloning the peptides under the control of a T7 promoter and then introducing this construct into an *N. tabacum* line whose chloroplast genome contained the gene for T7 RNA polymerase, which itself was controlled by an inducible theophylline riboswitch (Verhounig *et al*., 2010). Once plants had grown, watering them with theophylline allowed expression of the T7 polymerase and thus the peptides. A similar strategy was successfully applied for high expression of a synthetic astaxanthin pathway in *N. tabacum* (Agrawal *et al*., 2022).

The levels of vitamins required to modulate our two regulatory systems are very low: in the picomolar range for P_*METE*_‐NAC2 by B_12_ and nanomolar range for RS_*T4*_‐MRL1 by thiamine. Photosynthetic growth was more sensitive than transgene expression (compare Figure 2 to Figures 5 and 6), presumably due to the need to ensure sufficient levels of PsbD or RbcL to allow effective assembly of the photosynthetic complexes. This compares to micromolar levels needed for a chemical inducer such as IPTG, which would also be orders of magnitude more expensive than vitamins, an important consideration in the context of large‐scale production of an engineered strain of *Chlamydomonas*. Moreover, vitamins are benign compounds that have little or no effect on the overall transcriptome or proteome (Helliwell *et al*., 2014) and would have no harmful effects if released into the environment. It should be acknowledged that the systems are both NOT‐gates, that is, addition of the ligand switches off the gene. As a consequence, induction of transgene expression requires removal of the vitamins from the culture, which can be achieved by pelleting the cells and washing. While this is impractical for industrial‐scale production, one approach would be to grow the initial inoculum with vitamins to repress expression of the transgenes and ensure a healthy and dense culture. Then, since the concentrations of vitamins required are so low, the addition of this inoculum into a fresh medium in a large‐scale reactor would allow steady induction of expression due simply to dilution.

There is the potential to expand the regulatory toolkit we have developed by combinations of different genetic elements. In strain CSB195#1, which has both systems, the NAC2/*psbD* system is orthogonal to MRL1/*rbcL* and MRL1/*w1*. Although *Chlamydomonas* encodes just one TPR protein (NAC2), there are genes for another 13 PPR proteins and over 100 OPR (octatricopeptide repeat) proteins in the genome. There are also systems from other organisms that have been deployed, such as the utilization of recombinant PPR proteins (Shahar *et al*., 2021), which would increase the range of possibilities further. One limitation to this would be the need to identify additional elements equivalent to P_*METE*_ and RS_*T4*_ to regulate the expression of the nucleus‐encoded trans‐acting factors. An inducible and orthogonal approach to regulate gene expression in the *Chlamydomonas* used the alcohol‐inducible *AlcR‐*P*alcA* system, which originates from the filamentous fungus, *Aspergillus nidulans* and consists of two components, a regulatory protein, AlcR and an inducible promoter, P_*alcA*_ (Lee *et al*., 2018). This, however, suffered from the fact that there was measurable background expression without ethanol application, resulting in a narrow dynamic range. The application of completely different approaches to the use of small molecules, such as optogenetics using blue light (Chen *et al*., 2023), offers further expansion. It is clear that there are considerable opportunities to exploit *Chlamydomonas* as a sophisticated chassis for engineering biology both for fundamental studies and as a biotechnological host.

## Experimental procedures

### Cultivation of *Chlamydomonas* strains

*Chlamydomonas reinhardtii* strains (Table S1) were grown mixotrophically or heterotrophically in TAP media (Gorman and Levine, 1965) or photoautotrophically in HSM (Sueoka, 1960), supplemented with trace elements (Kropat *et al*., 2011), either on agar plates (2% w/v, Formedium Ltd, UK) or liquid media, at 25 °C under constant illumination (90 μmol photons/m^2^/s) or in low light (<10 μmol photons/m^2^/s; photosynthetic mutant strains only). Cultures set up in cell culture flasks (Nunc EasYFlask 25 cm^2^, filter closure, Thermo Scientific, UK) were grown in shaking incubators at 120 rpm (Infors Multitron, Infors UK Ltd), while cultures in 96‐well microplates (volume 200 μL) were grown in stationary incubators. The optical density of liquid cultures was measured using UV–VIS spectrophotometer (Thermo Scientific, UK), FLUROstar OPTIMA or CLARIOstar Plus plate readers (BMG Labtech, UK). Cell density was measured in C‐Chip haemocytometer slides (Cambridge Bioscience, UK) using the Rebel hybrid microscope (Discover Echo). When appropriate, cultures were supplemented with spectinomycin (200–300 μg/mL; Melford, UK), 5‐fluorocytosine (5 mg/mL; Fisher Scientific, UK), thiamine (1 nM–1 μM; Sigma–Aldrich, UK) or B_12_ (0.7 pM–1 μM; Sigma–Aldrich, UK), as detailed in the text.

### Plasmid design and assembly

Plasmids (Table S2) were generated using modular cloning methods. Constructs for transforming the nuclear genome were designed using parts from the *Chlamydomonas* MoClo toolkit (Crozet *et al*., 2018) and those for transforming the chloroplast genome from standard parts using the Start‐Stop Assembly method (Taylor *et al*., 2018) and included flanking regions for homologous recombination at the appropriate loci. Plasmids were assembled in chemically competent *E. coli* DH5α cells followed by selection on LB agar plates with respective antibiotics (carbenicillin, 100 μg/mL; tetracycline, 10 μg/mL; spectinomycin, 100 μg/mL). Plasmids were extracted and purified using Monarch® Miniprep Kit (NEB, UK) for small‐scale cultures or GenEluteTM HP Midiprep Kit (Sigma‐Aldrich, UK) for medium‐scale cultures. Plasmid concentrations were determined using a NanoDropTM spectrophotometer (Thermo Scientific, UK). All plasmids were verified by enzymatic digest with appropriate restriction enzymes and sequence identity was confirmed by Sanger sequencing (Azenta, UK).

### Transformation of *Chlamydomonas*

Nuclear transformation of the *mrl1‐5* and *nac2‐26* mutants of *Chlamydomonas* was carried out by electroporation as previously described (Mehrshahi *et al*., 2020) with the following modifications. Cells were grown in TAP media under <10 μmol m^2^/s illumination to a density of 1–5 × 10^6^ cells/mL, then harvested by centrifugation, before resuspension in 250 μL of GeneArt High Efficiency buffer (Thermo Scientific, UK), and then electroporation as described. After recovery in TAP containing 60 mM sucrose at 25 °C for 16–18 h under low light with shaking (120 rpm), cells were recovered and resuspended in 500 μL of liquid HSM, which was spread across three HSM agar plates. Plates were incubated at 25 °C under low light for 24 h and then transferred to continuous light (90 μmol photons/m^2^/s). Colonies appeared after 3–5 weeks of incubation, and restoration of photosynthetic competence was confirmed by restreaking on HSM.

Chloroplast transformation followed the protocol of Jackson *et al*. (2022) using bombardment with a Biolistics PDS‐1000/HE device (Bio‐Rad Laboratories, UK), with 1350 psi rupture discs (Bio‐Rad Laboratories). To reach homoplasmy, 16 colonies per construct were streaked onto fresh TAP agar plates, incubated for 7 days and then re‐streaked up to three times with successively higher concentrations of antibiotics. Genotypes of transformed strains were confirmed by using Phire Plant Direct PCR Kit (Thermo Scientific, UK) according to the manufacturer's protocol. Transgene integration was verified using one primer annealing to the transgene cassette and one annealing to the genome flanking the integration site (Table S3). Homoplasmy was verified using primers that specifically amplify the WT gene at the integration locus, such that no product was formed once all chloroplast genome copies contained the transgene.

### RNA extraction and RT‐qPCR

RNA isolation and RT‐qPCR were carried out as previously described (Bunbury *et al*., 2020) with the following modification, extraction was performed on liquid‐nitrogen frozen cell pellets prepared from cultures grown to late‐log phase of growth (5 mL). Expression levels of chloroplast genes were normalized to the nuclear genome‐encoded control gene *RACK1* (Mus *et al*., 2007).

### Mating of *Chlamydomonas*

Strains of the opposite mating types (mt+ and mt−) were first grown in a patch of around 1 cm × 3 cm cells each on a fresh TAP plate for 3–4 days. The strains were transferred to a smaller area of 1 cm × 2 cm on TAP N10 plates, which contained one‐tenth of the standard ammonium chloride concentration, and grown in high light (>200 μmol photons/m^2^/s) for 3–4 days. Each strain was resuspended in 2–5 mL of sterile water in Erlenmeyer flasks to achieve cell density between 0.5–2 × 10^7^ cells/mL and shaken (50 rpm) for at least in 30 min, before assessing the mobility of gametes under the microscope. The two strains were then mixed in the same flask with a cell ratio 1:1 and grown under high light (>200 μmol photons/m^2^/s) with gentle mixing. After 2–3 h, the flask was checked for the presence of cell aggregates, then cells were plated onto TAP agar plates. The plates were left up‐side‐down under high light for 12–14 h, wrapped with tin foil and incubated upside‐down at 25 °C for a further 14 days in darkness. Zygotes migrated inside the agar, whereas vegetative cells remained on the top. Vegetative cells were removed with a razor and the plate was treated with vapours of chloroform for 30 s. The zygote‐rich sections were cut out, transferred to a 15 mL Falcon tube containing 2 mL of TAP and incubated under high light without shaking. After 2 days, the culture was vortexed for 1–2 min, and 100–200 μL of the cell suspension was plated on TAP agar plates and incubated at 25 °C until colonies were formed.

### Fluorescence measurements

Samples of cultures in logarithmic phase of growth (200 μL) were transferred to 96‐well plates. Fluorescence was measured using CLARIOstarTM plate reader (BMG LabTech, UK). TAP medium was used as a blank. The fluorescence of mVenus (excitation 515 ± 10 nm, emission 550 ± 10 nm) and CFP (excitation 430 ± 20 nm, emission 480 ± 20 nm) were normalized to chlorophyll fluorescence (excitation 440 ± 9 nm, emission 680 ± 20 nm).

### Luciferase assay

Luciferase assays were performed using Nano‐Glo® Luciferase Assay System (Promega, UK). Samples of cultures in logarithmic phase of growth (1 mL) were centrifuged at 1000 ***g*** for 10 min, and pellets were resuspended in phosphate‐buffered saline (PBS) to OD_730 nm_ of 0.5. Samples (50 μL) were mixed with the lysis buffer (50 μL; 2 × Cell Culture Lysis Reagent Promega, UK, diluted in PBS) and incubated at room temperature for 10 min. Cell extracts (25 μL) were mixed with an equal volume of Nano‐Glo® Luciferase Assay Substrate solution. Luminescence was measured at 460 nm using a CLARIOstarTM plate reader after 3 min incubation.

### Casbene detection and quantification

To measure casbene production, strains of interest were cultured in a 20 mL TAP medium in Nunc flasks [with a 10% *n*‐dodecane overlay (Alfa Aesar, UK) added 4 days post‐inoculation]. After 8 days, cultures were centrifuged to separate the two phases and extract dodecane. To quantify casbene, 50 μL of the dodecane phase was mixed with an equal volume of hexane and analysed by GC–MS, using 250 μM *trans*‐caryophyllene as an internal standard. Casbene peak areas were determined from the chromatograms with mass ranges 91.0, 97.0, 107.0, 122.0 and 272.0 and normalized to caryophyllene.

## Accession numbers

Sequences of all plasmids, including the optimized coding sequences, have been deposited in NCBI and the accession numbers are given in Table S2.

## Author contributions

AG‐R, PMM, PM and AGS designed the research; AG‐R, PMM, KC, CS, DQZ, KG and PM performed the research; AG‐R, PMM, KC, KG, PM and AGS analysed the data; PMM, KC and AGS wrote the paper with input from all authors.

## Conflict of interest

The authors declare no conflict of interest.

## Supporting information


**Figure S1** PCR genotyping of METE_N#5, T4M#5 and MN_dc#1.
**Figure S2** Time course of the effect of vitamin supplementation on photosynthetic growth of *nac2‐26* and *mrl1‐5* complemented lines.
**Figure S3** Characterization of strains RSW.
**Figure S4** Construction and characterization of strains CSB193 and CSB195.
**Figure S5** Phototrophic and mixotrophic growth of CSB195#1 and CC‐1690.
**Figure S6** Activity and thiamine responsiveness of P*rrnS/5'rbcL* chimeric promoter‐5′UTR.
**Figure S7** Screening and characterization of CSB292 and CSB306 transformants.
**Figure S8** B_12_‐dependent regulation of *rbcL* in METE_M strain.
**Figure S9** Sequences and alignment of 158 bp genome fragments located upstream of *rbcL* and *wendy1* start codons.
**Figure S10** Genotyping of *JcCS* and *SpDxs* transformants.
**Table S1** List of *Chlamydomonas reinhardtii* strains used or generated in this study.
**Table S2** List of plasmids generated and used in this study.
**Table S3** Complementation of *nac2‐26* and *mrl1‐5* mutants with WT genes and their responsiveness to vitamin supplementation.
**Table S4** List of synthetic oligonucleotides used for quantitative RT‐PCR and genotyping in this study.

## Data Availability

The data supporting this study's findings are in the supplementary material.
