# Supplementary material for: Regulation of nucleus‐encoded trans‐acting factors allows orthogonal fine‐tuning of multiple transgenes in the chloroplast of Chlamydomonas reinhardtii
Source: Plant Biotechnol J. 2024 Dec 28;23(3):1005–18. doi: 10.1111/pbi.14557 (PMC11869193; doi:10.1111/pbi.14557)
Supplement: Supplementary file 1 — Figure S1 PCR genotyping of METE_N#5, T4M#5 and MN_dc#1. Figure S2 Time course of the effect of vitamin supplementation on photosynthetic growth of nac2‐26 and mrl1‐5 complemented lines. Figure S3 Characterization of strains RSW. Figure S4 Construction and characterization of strains CSB193 and CSB195. Figure S5 Phototrophic and mixotrophic growth of CSB195#1 and CC‐1690. Figure S6 Activity and thiamine responsiveness of PrrnS/5'rbcL chimeric promoter‐5′UTR. Figure S7 Screening and characterization of CSB292 and CSB306 transformants. Figure S8 B12‐dependent regulation of rbcL in METE_M strain. Figure S9 Sequences and alignment of 158 bp genome fragments located upstream of rbcL and wendy1 start codons. Figure S10 Genotyping of JcCS and SpDxs transformants. Table S1 List of Chlamydomonas reinhardtii strains used or generated in this study. Table S2 List of plasmids generated and used in this study. Table S3 Complementation of nac2‐26 and mrl1‐5 mutants with WT genes and their responsiveness to vitamin supplementation. Table S4 List of synthetic oligonucleotides used for quantitative RT‐PCR and genotyping in this study. [file PBI-23-1005-s001.docx]

**Regulation of nucleus-encoded trans-acting factors allows orthogonal fine-tuning of multiple transgenes in the chloroplast of *Chlamydomonas reinhardtii***

Pawel M. Mordaka, Kitty Clouston, Aleix Gorchs-Rovira, Catherine Sutherland, Daniel Q. Zhang, Katrin Geisler, Payam Mehrshahi, and Alison G. Smith

**Supplementary data**

**Table S1.** List of *Chlamydomonas reinhardtii* strains used or generated in this study. Transgenic strains from this study were generated by the introduction of the relevant plasmids as indicated. These are detailed in Table S2.

| **Strain** | **Genetic background or cross** | **Description** | **Reference** |
| --- | --- | --- | --- |
| CC-1690 mt+ | - | WT strain (21 gr) | Sager, 1955 |
| *nac2-26* mt+  (CC-4421) | CC-125 (137c) | Non-functional NAC2, does not accumulate *psbD* transcripts | Kuchka *et al*. 1989 |
| *mrl1-5* mt- | Jex4 mt- | Non-functional MRL1, does not accumulate *rbcL* transcripts | Johnson *et al*., 2011 |
| AaR-1 mt+ | *mrl1-5* mt+ | Photosynthesis restored by swapping *rbcL* promoter and 5’UTR for psaA | Gift from Katia Wostrikoff,  CNRS/UPMC |
| METE_N mt+ | *nac2-26* mt+ | *nac2-26* mutant complemented with plasmid pMETE_N, to restore photosynthesis. *METE* promoter results in B_12_ regulation of *NAC2* expression | This work |
| T4M mt- | *mrl1-5* mt- | *mrl1-5* mutant complemented with plasmid pT4M, to restore photosynthesis. THI4_N riboswitch means thiamine regulation of *MRL1* expression | This work |
| METE_M mt- | mrl1-5 mt- | *mrl1-5* mutant complemented with pMETE_N, to restore photosynthesis. *METE* promoter results in B_12_ regulation of *MRL1* expression | This work |
| MN_dc mt- | METE_N#5 mt+  T4M#5 mt- | Cross of METE_N#5 mt+ and T4M#5 mt-, selected for both transgenes and *nac2-26* and *mrl1-5* mutations. | This work |
| RSW | AaR-1 mt+  MN_dc#1 mt- | Cross of AaR-1 mt+ and MN_dc#1 mt- | This work |
| CSB193 | RSW#2 mt+ | Transformed with plasmid pCSB193 (Table S2), resulting in *psbD* promoter and 5’UTR swapped for those of *petA* | This work |
| CSB195 | RSW#2 mt+ | Transformed with plasmid pCSB195, codA.aadA marker looped-out, *psbD* promoter and 5’UTR swapped for those of *psaA* | This work |
| CSB292 | CSB195#1 | Transformed with plasmid pCSB292, expression of *Nluc* (thiamine regulation) and *mVenus.ME* (B_12_ regulation) from *psbA* locus | This work |
| CSB306 | CSB195#1 | Transformed with plasmid pCSB306, expression of *mVenus.ME* (thiamine regulation) and *Nluc* (B_12_ regulation) from *psbA* locus | This work |
| CSB178  CSB180  CSB181  CSB182  CSB183  CSB186  CSB200 | RSW#2 mt+ | Transformed with plasmids pCSB178, pCSB180, pCSB181, pCSB182, pCSB183, pCSB186 and pCSB200, expression of *Nluc* from *psbH* locus, using different promoters and 5’UTRs | This work |
| CSB339  CSB340  CSB341 | CSB195#1 | Transformed with plasmids pCSB339, pCSB340, pCSB341 and pCSB342, expression of *CS-mVenus* from *psbH* locus using different promoters and 5’UTRs | This work |
| CSB342 | CSB195#1 | Transformed with plasmid pCSB342, expression of *CS-mVenus* from *psbA* locus, using *wendy1* promoter and 5’UTR | This work |
| CSB353 | CSB195#1 | Transformed with plasmid pCSB353, expression of *CS-mVenus* (B_12_ regulation) and *DXS-CFP* (thiamine regulation) from *psbA* locus | This work |
| CSB354 | CSB195#1 | Transformed with plasmid pCSB354, expression of *CS-mVenus* (thiamine regulation) and *DXS-CFP* (B_12_ regulation) from *psbA* locus | This work |

**Table S2.** List of plasmids generated and used in this study. For level 2 plasmids assembled by Golden Gate cloning, individual level 1 plasmid have been highlighted with alternating shading. Sequences are available in the GenBank database (www.ncbi.nlm.nih.gov/genbank/) with the appropriate accession numbers. METE promoter-5’UTR (Helliwell *et al*., 2014); THI4_4N RS = modified THI4 riboswitch (Mehrshahi *et al*., 2020) AR = chimeric HSP70-RBCS2 promoter (Schroda *et al*., 2002); LHA = left homology arm for integration into chloroplast genome; RHA = right homology arm; HA = HA-tag; CA1 3’UTR = carbonic anhydrase1 terminator (Geisler *et al*., 2021)

| **Plasmid** | **Backbone** | **Level** | **Description** | **Accession number** |
| --- | --- | --- | --- | --- |
| pMETE_N | pICH47742 | L1 | METE promoter-METE’5UTR-NAC2-HA-CA1 3’UTR | PP639714 |
| pT4M | pICH47742 | L1 | AR promoter-THI4_4N RS-MRL1-HA-CA1 3’UTR | PP639715 |
| pMETE_M | pICH47742 | L1 | METE promoter-METE’5UTR-MRL1-HA-CA1 3’UTR | PP639716 |
| pPM900 | - |  | Empty level 0/2 acceptor plasmid | MT361981 |
| pCSB19 | - |  | Empty level 2 acceptor plasmid | PP746763 |
| pCSB193 | pPM900 | L2 | LHA(yfc2)-DR-PatpA-5’atpA-codA.aadA-TrbcL-DR-PpetA-5’petA-RHA(psbD) | PP746764 |
| pCSB195 | pPM900 | L2 | LHA(yfc2)-DR-PatpA-5’atpA-codA.aadA-TrbcL-DR-PpsaA-5’psaA-RHA(psbD) | PP746765 |
| pCSB292 | pCSB19 | L2 | LHA(psbA)-PrbcL-5’rbcL-Nluc-TatpB-PpsbD-5’psbD-mVenus.ME-TrbcL-PatpA-5’atpA-codA.aadA-TrbL-RHA(psbA) | PP746766 |
| pCSB306 | pCSB19 | L2 | LHA(psbA)-PrbcL-5’rbcL-mVenus.ME-TatpB-PpsbD-5’psbD-Nluc-TrbcL-PatpA-5’atpA-codA.aadA-TrbL-RHA(psbA) | PP746767 |
| pCSB339 | pCSB19 | L2 | LHA(psbH)-PrbcL-5’rbcL-CS-mVenus.ME-TrbcL-PatpA-5’atpA-codA.aadA-TrbcL-RHA(psbH) | PP746768 |
| pCSB340 | pCSB19 | L2 | LHA(psbH)-Pwendy1-5’wendy1-CS-mVenus.ME-TrbcL-PatpA-5’atpA-codA.aadA-TrbcL-RHA(psbH) | PP746769 |
| pCSB341 | pCSB19 | L2 | LHA(psbH)-PpsbD-5’psbD-CS-mVenus.ME-TrbcL-PatpA-5’atpA-codA.aadA-TrbcL-RHA(psbH) | PP746770 |
| pCSB342 | pCSB19 | L2 | LHA(psbA)-Pwendy1-5’wendy1-CS-mVenus.ME-TrbcL-PatpA-5’atpA-codA.aadA-TrbcL-RHA(psbA) | PP746771 |
| pCSB353 | pCSB19 | L2 | LHA(psbA)-PpsbD-5’psbD-CS-mVenus.ME-TrbcL-PatpA-5’atpA-codA.aadA-TrbcL-Pwendy1-5’wendy-DXS-CFP-TatpB-RHA(psbA) | PP746772 |
| pCSB354 | pCSB19 | L2 | LHA(psbA)-Pwendy1-5’wendy1-CS-mVenus.ME-TrbcL-PatpA-5’atpA-codA.aadA-TrbcL-PpsbD-5’psbD-DXS-CFP-TatpB-RHA(psbA) | PP746773 |
| pCSB178 | pPM900 | L2 | LHA(psbH)-‘ag’ spacer-Nluc-TatpB-PatpA-5’atpA-codA.aadA-TrbcL-RHA(psbH) | PP746774 |
| pCSB180 | pPM900 | L2 | LHA(psbH)-‘ab’ spacer-rbcL 5’UTR-Nluc-TatpB-PatpA-5’atpA-codA.aadA-TrbcL-RHA(psbH) | PP746775 |
| pCSB181 | pPM900 | L2 | LHA(psbH)-P16S-psaA 5’UTR-Nluc-TatpB-PatpA-5’atpA-codA.aadA-TrbcL-RHA(psbH) | PP746776 |
| pCSB182 | pPM900 | L2 | LHA(psbH)-PpsaA-psaA 5’UTR-Nluc-TatpB-PatpA-5’atpA-codA.aadA-TrbcL-RHA(psbH) | PP746777 |
| pCSB183 | pPM900 | L2 | LHA(psbH)-PrbcL-rbcL 5’UTR-Nluc-TatpB-PatpA-5’atpA-codA.aadA-TrbcL-RHA(psbH) | PP746778 |
| pCSB186 | pPM900 | L2 | LHA(psbH)-P16S-rbcL 5’UTR-Nluc-TatpB-PatpA-5’atpA-codA.aadA-TrbcL-RHA(psbH) | PP746779 |
| pCSB200 | pPM900 | L2 | LHA(psbH)-PpsbD-psbD 5’UTR-Nluc-TatpB-PatpA-5’atpA-codA.aadA-TrbcL-RHA(psbH) | PP746780 |

**Table S3.** **Complementation of *nac2-26* and *mrl1-5* mutants with WT genes and their responsiveness to vitamin supplementation**. Transformants of each mutant were selected by their ability to grow on minimal medium (HSM), demonstrating restoration of photosynthesis. They were then tested for their responsiveness to 700 pM B_12_ (*nac2-26* transformants) or 1000 nM thiamine (*mrl1-5* transformants), which would result in repression of the NAC2 or MRL1 respectively, and thus they could no longer grow on HSM.

| **Plasmid** | **Strain** | **mt+** | **mt-** | **Responsiveness to vitamin supplementation** |
| --- | --- | --- | --- | --- |
| pMETE_N | *nac2-26* | 20 | 15 | 65% |
| pT4M | *mrl1-5* | 21 | 20 | 95% |

**Table S4.** List of synthetic oligonucleotides used for quantitative RT-PCR and genotyping in this study.

| **Name** | **Sequence** | **Description** |
| --- | --- | --- |
| **pT4M and pMETE_N transformation** | | |
| AG.236 | TTGAAGACATGGAGGTAGGTCAGGACCAGAGCCT | P_METE_ forward primer |
| AG.266 | CAGCGACTGATGGCATTTAGC | NAC2 CDS reverse primer |
| AG.238 | TTGAAGACATTACTGAGCTGTCGCATAGATCGC | RS_T4_ forward primer |
| AG.241 | TGGTGTCTCGACTAGGGTACTC | MRL1 reverse primer |
| mt+_F | ATGCCTATCTTTCTCATTCT | mt+ (fus1) forward primer |
| mt+_R | GCAAAATACACGTCTGGAAG | mt+ (fus1) reverse primer |
| mt-_F | ATGGCCTGTTTCTTAGC | mt- (mid) forward primer |
| mt-_R | CTACATGTGTTTCTTGACG | mt- (mid) reverse primer |
| **qPCR primers** | | |
| AG.601 | GCTGCTGTAGCTGCTGAATC | *rbcL* forward primer |
| AG.602 | TAAGTCGATTGGGTAAGCTACGTAAG | *rbcL* reverse primer |
| AG.603 | GCATTAGGTGGTTGGTTAACTGGTAC | *psbD* forward primer |
| AG.604 | AGTTGACACCAACGAGTGAAATCAC | *psbD* reverse primer |
| ON_56.F | CGTCTGTGGGACCTGAACAC | *RACK1* forward primer |
| ON_57.R | GCTCGCCAATGGTGTACTTG | *RACK1* reverse primer |
| **Generation of CSB193 and CSB195** | | |
| oligoCSB145 | GCGCAGATCAGTTGGAAGA | *codA.aadA* cassette loop-out forward primer |
| oligoCSB492 | CGGTATTCGGAATCTTGCAC | cassette integration forward primer |
| oligoCSB493 | TTCGTGTGGTTGGTCTTGAG | *psbD* locus, homoplasmy, cassette integration and codA.aadA loop-out reverse primer |
| oligoCSB496 | TGCACAAAGCAGTTCTAGTCC | *psbD* locus, homoplasmy forward primer |
| **Generation of CSB292, CSB306, CSB342** | | |
| oligoCSB145 | GCGCAGATCAGTTGGAAGA | cassette integration forward primer |
| oligoCSB591 | ggtaggttctgtcactgac | *psbA* locus, homoplasmy and cassette integration reverse primer |
| oligoCSB596 | TTCCGAAACGGTGGTTATTC | *psbA* locus, homoplasmy forward primer |
| **Generation of CSB178,180-186 and CSB200** | | |
| oligoCSB349 | GTCATTGCGAAAATACTGGTGC | *psbH* locus, homoplasmy and cassette integration forward primer |
| oligoCSB352 | AGTATTACCAGGCTGGGGTACA | *psbH* locus, homoplasmy reverse primer |
| oligoCSB415 | CCGTTTTCACCTGATAAAACAA | cassette integration forward primer |
| **Generation of CSB339-341** | | |
| oligoCSB145 | GCGCAGATCAGTTGGAAGA | cassette integration forward primer |
| oligoCSB349 | GTCATTGCGAAAATACTGGTGC | *psbH* locus, homoplasmy forward primer |
| oligoCSB352 | AGTATTACCAGGCTGGGGTACA | *psbH* locus, homoplasmy and cassette integration reverse primer |
| **Generation of CSB353 and CSB354** | | |
| oligoCSB412 | GATCACCCCAAACTGTTGGT | cassette integration reverse primer |
| oligoCSB591 | ggtaggttctgtcactgac | *psbA* locus, homoplasmy reverse primer |
| oligoCSB596 | TTCCGAAACGGTGGTTATTC | *psbA* locus, homoplasmy and cassette integration forward primer |

**Figure S1. PCR genotyping of METE_N#5, T4M#5 and MN_dc#1.** (a) Amplification of the P_METE_-NAC2 cassette with primers AG.236 and AG.266 (Table S4). DNA ladder - HyperLadderTM 1kb (Bioline). Expected product: 1084 bp. (b) Amplification of the RS_T4_-MRL1 cassette with primers AG.238 and AG.241. DNA ladder - HyperLadderTM 1kb (Bioline). Expected product: 1486 bp. (c) Genotyping of MN_dc#1. Amplification of P_METE_-NAC2 and RS_T4_-MRL1 cassettes (left) and mating type locus with primers mt+_F/mt+_R and mt-_F/mt-_R (right). Expected bands: mt+ (516 bp), mt- (622 bp). DNA ladders - HyperLadderTM 1kb (left) and HyperLadderTM 100bp (right). WT = wild type; NT = no template.

**Figure S2. Time course of the effect of vitamin supplementation on photosynthetic growth of *nac2-26* and *mrl1-5* complemented lines.** Cells were grown in photoautotrophic HSM media and growth was monitored by measuring optical density at 730 nm (OD_730_). (a) Effect of increasing concentrations of B_12_ on growth of METE_N#8 and METE_N#14. (b) Effect of increasing concentrations of thiamine on growth of T4M#11 and T4M#14.

**Figure S3. Characterisation of strains RSW.** (a) PCR genotyping of progeny obtained from crossing Aar-1 (mt+) with MN_dc#1 (mt-). Amplification of the RS_T4_-MRL1 cassette with primers AG.238 and AG.241 (top left) and P_METE_-NAC2 cassette with primers AG.236 and AG.266 (top right). Amplification of the mt+ locus with primers mt-_F and mt-_R (bottom). DNA ladder - HyperLadderTM 1kb (Bioline) and HyperLadderTM 100bp (Bioline). WT = wild type; NT = no template. (b) Effect of increasing concentrations of thiamine (left) or B_12_ (right) on photosynthetic growth in HSM as measured by OD_730_ of RSW#2. Error bars represent standard deviations (n = 3).

**Figure S4. Construction and characterisation of strains CSB193 and CSB195.** (a) PCR genotyping of individual transformants of RSW#2 with plasmids pCSB193 and pCSB195. Transformants were re-streaked 3 times on increasing concentration of antibiotics and single colonies were genotyped using Phire Plant Direct PCR Kit (Thermo Scientific, UK). Multiplex PCR was performed with three primers: oligoCSB492 (annealing to the recombinant cassette upstream of the swapped *psbD* promoter), oligoCSB493 (annealing to the *psbD* coding sequence downstream of the right homology arm) and oligoCSB496 (annealing to the *psbD* promoter). Integration of the recombinant cassette, expected products: CSB193 – 1785 bp, CSB195 – 1384 bp. Presence of the parental strain DNA (RSW#2) – 1210 bp. NT = no template. DNA ladder - HyperLadderTM 1kb (Bioline). (b) Photosynthetic growth of CSB193 (#3, #5, #6, #11 and #12) and CSB195 transformants (#1, #2, #3, #4, #11, #12, #13) and the parental strain RSW#2 (n=2) in HSM as measured by OD730. Error bars represent standard deviations.

**Figure S5. Phototrophic and mixotrophic growth of CSB195#1 and CC-1690.** CSB195#1 and a wild-type strain CC-1690 for comparison were grown in HSM (photosynthetic conditions) and TAP (mixotrophic conditions). Growth was measured by OD730 (left) and cell counting using Neubauer Improved C-Chip Disposable Haemocytometer (NanoEnTek) with Echo Rebel Hybrid Microscope (right). Error bars represent standard deviations (n=3).

**Figure S6. Activity and thiamine responsiveness of *PrrnS/5’rbcL* chimeric promoter-5’UTR.** Relative activity of luciferase in the transgenic lines CSB181 (*PrrnS/5’psaA*) and CSB186 (*PrrnS/5’rbcL*) . Independent homoplasmic transformants of each construct (n=4) were grown in TAP medium and samples were harvested on day 4. To test regulation of reporter expression, cultures were supplemented with thiamine (1 μM).

**Figure S7. Screening and characterisation of CSB292 and CSB306 transformants.** (a) Confirmation of the reporter cassette integration (top) and homoplasmy test (bottom) for transformants with plasmids pCSB292 (left) and pCSB306 (right). Single colonies were genotyped using Phire Plant Direct PCR Kit. Expected products: reporter cassette integration using oligoCSB145 and oligoCSB591 – 1360 bp, homoplasmy test using oligoCSSB596 and oligoCSB591 – 1102 bp. (b) Relative luciferase activity (left) and normalised mVenus fluorescence (right) of homoplasmic lines CSB292 (top) and CSB306 (bottom). Repression of reporter expression was tested by supplementation with thiamine (1 µM) and B_12_ (10 nM). (c) Time course measurement of mVenus expression in CSB292#17 and CSB306#2. Error bars represent standard deviations (n=3).

**Figure S8. B_12_-dependent regulation of *rbcL* in METE_M strain.** (a) Schematic of construct pMETE_M used to complement the *mrl1-5* strain, where MRL1 is controlled by the METE promoter and 5’UTR. (b) Effect of increasing concentrations of B_12_ on growth of three independent METE_M transformants (left). For comparison, effect of increasing concentrations of thiamine on growth of three independent T4M lines was tested alongside. Cells were grown in photoautotrophic HSM media and growth was monitored by measuring optical density at 730 nm (OD_730_).

>rbcL

TGATAAGACAAGTACATAAATTTGCTAGTTTACATTATTTTTTATTTCTAAATATATAATATATTTAAATGTATTTAAAAT
TTTTCAACAATTTTTAAATTATATTTCCGGACAGATTATTTTAGGATCGTCAAAAGAAGTTACATTTATTTATATAAATG

>wendy1

ATTAGCATGTTTTTTCCTAAAATATATTTATTTGACATAAATATATTTATGTGATATAATATATTTAAATGTATTTAAAAT
TTTTCAACAATTTTTAAATTATATTTCCGGACAGATTATTTTAGGATCGTCAAAAGAAGTTACATTTATTTAGAACTATG

rbcL --TGATAAGACAAGTACATAAATTTGCTAGTTTACATTATTTTTTATTTCTAAATATATA 58

wendy1 ATTAGCATGTTTTTTCCTAAAATATATT--TATTTGACATAAATATATTTATGTGATATA 58

* * * * * **** * * * * ** * ** *****

rbcL ATATATTTAAATGTATTTAAAATTTTTCAACAATTTTTAAATTATATTTCCGGACAGATT 118

wendy1 ATATATTTAAATGTATTTAAAATTTTTCAACAATTTTTAAATTATATTTCCGGACAGATT 118

************************************************************

rbcL ATTTTAGGATCGTCAAAAGAAGTTACATTTATTTATATAAATG 161

wendy1 ATTTTAGGATCGTCAAAAGAAGTTACATTTATTTAGAACTATG 161

*********************************** * ***

**Figure S9. Sequences and alignment of 158 bp genome fragments located upstream of *rbcL* and *wendy1* start codons.** Start codons are highlighted in green, 5’UTR of *rbcL* (according to transcriptomic data from Gallaher et al., 2018) is highlighted in grey. Alignment was generated using clustalo (1.2.4) on ebi.ac.uk (accessed on 7^th^ May 2024).

**Figure S10. Genotyping of *JcCS* and *SpDxs* transformants.** (a) Confirmation of the reporter cassette integration (top gel) and homoplasmy test (bottom gel) for lines transformed with the different versions of *JcCS* cassette (Table S2) and (b) the *JcCS* and *SpDXS* cassette. Primers used for amplification are listed on the right and sizes of the expected products on left. CSB195#1 was used as a control. DNA ladder - HyperLadderTM 1kb (Bioline).

**REFERENCES**

[Gallaher, S.D., Fitz-Gibbon, S.T., Strenkert, D., Purvine, S.O., Pellegrini, M., and Merchant, S.S. (2018) *High-throughput sequencing of the chloroplast and mitochondrion of Chlamydomonas reinhardtii to generate improved de novo assemblies, analyze expression patterns and transcript speciation, and evaluate diversity among laboratory strains and wild isolates*. *Plant J.*, **93**, 545–565.](http://paperpile.com/b/LzHvhn/ttdY)

[Geisler, K., Scaife, M.A., Mordaka, P.M., Holzer, A., Tomsett, E.V., Mehrshahi, P., et al. (2021) *Exploring the impact of terminators on transgene expression in Chlamydomonas reinhardtii with a synthetic biology approach*. *Life*, **11**, 964.](http://paperpile.com/b/LzHvhn/qHsV)

[Helliwell, K.E., Scaife, M.A., Sasso, S., Araujo, A.P.U., Purton, S., and Smith, A.G. (2014) *Unraveling vitamin B12-responsive gene regulation in algae*. *Plant Physiol.*, **165**, 388–397.](http://paperpile.com/b/LzHvhn/6RN6)

[Johnson, X. (2011) *Manipulating RuBisCO accumulation in the green alga, Chlamydomonas reinhardtii*. *Plant Mol. Biol.*, **76**, 397–405.](http://paperpile.com/b/LzHvhn/eBYq)

[Kuchka, M.R., Goldschmidt-Clermont, M., van Dillewijn, J., and Rochaix, J.D. (1989) *Mutation at the Chlamydomonas nuclear NAC2 locus specifically affects stability of the chloroplast psbD transcript encoding polypeptide D2 of PS II*. *Cell*, **58**, 869–876.](http://paperpile.com/b/LzHvhn/RklN)

[Mehrshahi, P., Ginnie Trinh D, Rovira, A.G., Sayer, A., Llavero-Pasquina, M., Sin, M.L.H., et al. (2020) *Development of Novel Riboswitches for Synthetic Biology in the Green Alga Chlamydomonas*. *ACS Synthetic Biology*.](http://paperpile.com/b/LzHvhn/S2MN)

[Sager, R. (1955) *Inheritance in the Green Alga Chlamydomonas Reinhardi*. *Genetics*, **40**, 476–489.](http://paperpile.com/b/LzHvhn/i2iT)

[Schroda, M., Beck, C.F., and Vallon, O. (2002) *Sequence elements within an HSP70 promoter counteract transcriptional transgene silencing in Chlamydomonas*. *Plant J.*, **31**, 445–455.](http://paperpile.com/b/LzHvhn/5yjh)
